# Antagonism of Betulinic Acid on LPS-Mediated Inhibition of ABCA1 and Cholesterol Efflux through Inhibiting Nuclear Factor-kappaB Signaling Pathway and miR-33 Expression

**DOI:** 10.1371/journal.pone.0074782

**Published:** 2013-09-25

**Authors:** Guo-Jun Zhao, Shi-Lin Tang, Yun-Cheng Lv, Xin-Ping Ouyang, Ping-Ping He, Feng Yao, Wu-Jun Chen, Qian Lu, Yan-Yan Tang, Min Zhang, Yuchang Fu, Da-Wei Zhang, Kai Yin, Chao-Ke Tang

**Affiliations:** 1 Institute of Cardiovascular Research, Key Laboratory for Atherosclerology of Hunan Province, University of South China, Hengyang, Hunan, China; 2 Department of Histology and Embryology, University of South China, Hengyang, Hunan, China; 3 School of Nursing, University of South China, Hengyang, Hunan, China; 4 Department of Nutrition Sciences, University of Alabama at Birmingham, Birmingham, Alabama, United States of America; 5 Department of Pediatrics and Group on the Molecular and Cell Biology of Lipids, University of Alberta, Edmonton, Alberta, Canada; University of Barcelona, Spain

## Abstract

ATP-binding cassette transporter A1 (ABCA1) is critical in exporting cholesterol from macrophages and plays a protective role in the development of atherosclerosis. The purpose of this study was to investigate the effects of betulinic acid (BA), a pentacyclic triterpenoid, on ABCA1 expression and cholesterol efflux, and to further determine the underlying mechanism. BA promoted ABCA1 expression and cholesterol efflux, decreased cellular cholesterol and cholesterol ester content in LPS-treated macrophages. Furthermore, we found that BA promoted ABCA1 expression via down-regulation of miR-33s. The inhibition of LPS-induced NF-κB activation further decreased miR-33s expression and enhanced ABCA1 expression and cholesterol efflux when compared with BA only treatment. In addition, BA suppressed IκB phosphorylation, p65 phosphorylation and nuclear translocation, and the transcription of NF-κB-dependent related gene. Moreover, BA reduced atherosclerotic lesion size, miR-33s levels and NF-κB activation, and promoted ABCA1 expression in apoE^−/−^ mice. Taken together, these results reveal a novel mechanism for the BA-mediated ABCA1 expression, which may provide new insights for developing strategies for modulating vascular inflammation and atherosclerosis.

## Introduction

Atherosclerotic cardiovascular disease is one of the major causes of death in developed countries. Lipid accumulation, foam cell formation and inflammation are recognized as major features of atherosclerosis [1]. Inflammatory mechanisms play a central role in the pathogenesis and in the progression of each characteristic lesion/stage of atherogenesis and in the associated thrombotic complications [2]. Evidence of a link between inflammation and lipid metabolism is provided by studies showing dyslipidemia and insulin resistance during acute inflammation, as occurs in septic shock or trauma [3].

It has been demonstrated that the ATP binding cassette transporter A1 (ABCA1) plays a key regulatory role in cellular cholesterol metabolism. ABCA1 knockout cells or animals show reduced cholesterol efflux. ABCA1 is a rate-limiting factor of HDL assembly and its levels are regulated by transcriptional and post-transcriptional factors [4], [5]. We and others have shown that ABCA1 expression is reduced by exposure to inflammatory stimuli including IL-1β, TNF-α, IFN-γ and LPS in a nuclear factor-κB (NF-κB) dependent pathway [6]–[8]. Thus, Thus, inhibition of the NF-κB pathway may be a strategy for attenuating the downregulation of ABCA1 induced by an inflammatory stimulus although the exact mechanisms are not fully understood.

Betulinic acid (BA) is a naturally occurring triterpenoid widely distributed throughout the plant kingdom. BA has a wide range of pharmacological properties such as anti-cancer, malarial, retroviral and inflammatory properties [9]. BA also has an anti-obese potential through modulation of fat and carbohydrate metabolism [10]. One anti-inflammation mechanism of BA is attributed to its effect on NF-κB activation through inhibition of IκB kinase and p65 phosphorylation [11]. Given that ABCA1 expression is suppressed by inflammatory stimulus in a NF-κB dependent manner, it is possible that BA may regulate ABCA1 levels. Thus, we investigated the possible effects and mechanisms of BA on ABCA1 function *in vitro* and *in vivo*. Our results revealed that BA promotes ABCA1 expression and ABCA1-dependent cholesterol efflux via down-regulation of microRNA (miR)-33s. The suppressive effect of BA on the levels of miR-33s was mediated through the inhibition of NF-κB activation pathway.

## Materials and Methods

### Cell Culture

Human THP-1 macrophages were cultured in RPMI-1640 supplemented with 0.1% nonessential amino acids, penicillin (100 U/mL), streptomycin (100 µg/mL) and 20% fetal bovine serum (FBS). Cells were incubated at 37°C in a humidified atmosphere of 5% CO_2_. After 3 to 4 days, THP-1 cells were treated with phorbol-12-myristate- 13-acetate (PMA, 160 nmol/L; Sigma Chemical Co) for 24 hours, and then the medium was replaced with serum-free medium containing ox-LDL (50 µg/ml) for 48 hours to fully differentiate THP-1 to macrophages before their use in experiments.

### RNA Extraction and Real-time PCR

Total RNA was extracted using TRIzol reagent in accordance with the manufacturer’s instructions and cDNA fragments were generated by reverse transcription. Real-time quantitative PCR was performed on a Roche Light Cycler Run 5.32 Real-Time PCR System using SYBR Green detection chemistry. The sequences of the real-time PCR primers are as follows: human ABCA1, 5′-GGTTTG GAGATGGTTATACAATAGTTGT-3′ and 5′-CCCGGAAACGCAAGTCC-3′; mouse ABCA1, 5′-CGTTTCCGGGAAGTGTCCTA-3′ and 5′-GCTAGAGATGACAAGG AGGATGGA-3′; human SREBP-2, 5′-AGGAGAACATGGTGCTGA-3′ and 5′-TAA AGGAGAGGCACAGGA-3′. Melt curve analyses of all real-time PCR products were performed and shown to produce a single DNA duplex. Quantitative measurements were determined using the △△Ct method and the expression of β-actin was used as the internal control [12]. For microRNA assays, total RNA was extracted from cells using miRVana miRNA isolation kit (Life Technologies) and reverse transcribed by standard real-time qPCR (Applied Biosystems). Mir-33a/b was quantified using the TaqMan microRNA assay kit for has-miR-33a/b (Applied Biosystems), with U6 RNA used as an internal control [13].

### miR-33 and Anti-miR-33 Transfection

Human THP-1 cells were transfected with 40 nM miRIDIAN miRNA mimics (miR-33a/b) or with 60 nM miRIDIAN miRNA inhibitors (anti-miR-33a/b) (Dharmacon) utilizing Oligofectamine (Invitrogen). An equal concentration of a non-targeting control mimics sequence (Con miR) or inhibitor negative control sequence (Con Inh) was used as controls for non-sequence-specific effects in miRNA experiments. Verification of miR-33 overexpression and knockdown was determined using RT-PCR, as described above [14].

### Cholesterol Efflux Assays

Cholesterol efflux experiments were performed as previously described [15]. In brief, THP-1 macrophage derived foam cells (5×10^5^ cells) were seeded into 12-well plates. On day 3, cells were labeled with 0.5 µCi/ml of [^3^H]-cholesterol (PerkinElmer, Waltham, MA) in media containing 0.2% bovine serum albumin (BSA) for 24 h. The next day, cells were washed with fresh media and then treated as indicated in the figures. The cells were washed again with PBS and incubated in the presence of apoA-I (10 µg/mL) for 24 h. Medium and cell-associated [^3^H] cholesterol were then measured via liquid scintillation counting. Percent efflux was calculated by the following equation: [total media counts/(total cellular counts+total media counts)]×100%.

### High Performance Liquid Chromatography Assays

High performance liquid chromatography (HPLC) analysis was conducted as described previously [16]. Briefly, cells were washed with PBS for three times. The appropriate volume (usually 1 ml) of 0.5% NaCl was added to about 50–200 µg cellular proteins per ml. Cells were sonicated using an ultrasonic processor for 2 min. The protein concentration in cell solution was measured using BCA kit. An equal volume of freshly prepared cold (−20°C) KOH in ethanol (150 g/L) was added. The cell lysate was repeatedly vortexed until clear. An equal volume of hexane: isopropanol 3∶2 (v/v) was then added. The mixture was vortexed for 5 min, followed by centrifugation at 800×g (15°C for 5 min). The extraction procedure was repeated twice. 0.1 ml of aliquot cell solution (containing 5–20 µg protein) was used to measure the free cholesterol, and another aliquot for total cholesterol detection. Free cholesterol was dissolved in isopropanol (1 mg cholesterol/ml) and stored at −20°C as stock solution. Cholesterol standard calibration solution ranging from 0 to 40 µg of cholesterol per ml was obtained by diluting the cholesterol stock solution in the same cell lysed buffer.

0.1 ml of each sample (cholesterol standard calibration solutions, or cell solutions) was supplemented with 10 µl of reaction mixture including 500 mM MgCl_2_, 500 mM Tris–HCl (pH 7.4), 10 mM dithiothreitol, and 5% NaCl. 0.4 unit of cholesterol oxidase in 10 µl 0.5% NaCl was added to each tube for free cholesterol determination, or 0.4 unit of cholesterol oxidase plus 0.4 unit of cholesterol esterase for total cholesterol measurement. The total reaction solution in each tube was incubated at 37°C for 30 min, and then 100 µl of methanol:ethanol (1∶1) was added to stop the reaction. Each solution was kept cold for 30 min to allow protein precipitation, and then centrifuged at 1500 rpm for 10 min at 15°C. 10 µl of supernatant was applied onto a System Chromatographer (PerkinElmer Inc.) including a PerkinElmer series 200 vacuum degasser, a pump, a PerkinElmer series 600 LINK, and a PerkinElmer series 200 UV/vis detector and a Disovery C-18 HLPC column (Supelco Inc.). The column was eluted using isopropanol:n-heptane:acetonitrile (35∶13∶52) at a flow rate of 1 ml/min for 8 min. Absorbance at 216 nm was monitored. Data were analyzed with TotalChrom software from PerkinElmer.

### Cytokine ELISA

Cells were plated in 6-well plates and treated as described above [17]. Culture supernatants were collected and stored at −20°C until analysis. The concentrations of TNF-α, IL-6 and IL-1β in cell culture supernatants were measured by enzyme-linked immunosorbent assay (ELISA) (DuoSet ELISA Development System, R&D Systems, Abingdon, UK) following the manufacturer’s instructions. The serum levels of TNF-α, IL-6 and IL-1β assay were also measured using specific ELISA Kits. The cytokine standards were used to generate standard curves. Quantitative determinations in three different experiments were performed.

### Western Blot Analysis

Cells or murine tissues were harvested and protein extracts (for ABCA1, β-actin, IκBα and phospho-IκBα) and nuclear extracts (for histone H1, NF-κB p65 and phospho-NF-κB p65) were prepared as previously described [18]. The proteins (20 µg of lysates) were then loaded on 8% SDS-polyacrylamide electrophoresis gel, electrophoresed for 2 hours at 100 V in gel running buffer, and then transferred to polyvinylidene fluoride (PVDF) membranes. The primary antibodies used were anti- ABCA1, β-actin, histone H1, NF-κB p65, NF-κB p65 (phospho S536), IκBα and phospho-IκBα antibodies. (Santa Cruz, CA, USA). The proteins were visualized using a chemiluminescence method (enhanced chemiluminescence Plus Western Blotting Detection System; Amersham Biosciences, Foster City, CA).

### Mice and Treatments

Six-week old male apoE^−/−^ mice were obtained from Laboratory Animal Center of Peking University, China. All mice were fed a chow diet. At 8 weeks of age, apoE^−/−^ mice were randomly divided into several groups (n = 15 per group). The LPS group was challenged intraperitoneally (i.p.) with LPS (2.5 mg/kg body wt) in 200 µL of PBS once every week. The normal group was given an equivalent volume of PBS. Mice in the BA group were intragastrically administered with BA (50 mg/kg body wt) everyday and continued for 8 weeks on the bases of LPS challenge. At week 16, blood and tissues were collected from euthanized mice for further analysis.

The investigation followed the Guide for the Care and Use of Laboratory Animals published by the US National Institutes of Health (NIH publication no. 85-23, revised in 1996) and Care and Use guidelines for experimental animals of University of South China. The study protocol was approved by the Animal Ethics Committee of University of South China. All surgery was performed under sodium pentobarbital anesthesia, and efforts were made to minimize suffering.

### Lipid Analyses

Mice were fasted overnight and euthanized, and blood samples were obtained from the retro-orbital plexus. Triglyceride (TG), total cholesterol (TC), and HDL-C were determined by commercially enzymatic methods (test kits, Shanghai Rongsheng Biotech Inc. Shanghai, China).

### Evaluation of Aortic Lesions

Hearts and proximal aortas were removed and fixed. Hearts were cut directly under and parallel to the leaflet, and the upper portions were embedded in OCT medium and kept at 4°C. Eight µm thick sections were cut through the aortic sinus. Twenty sections per animal were stained for lipids with Oil-red O and counter-stained with Gill III hematoxylin (Sigma). Lesion areas were quantified with IMAGEPRO PLUS (Media Cybnetics, Silver Spring, MD), and data were expressed as lesion size ± SEM.

The aorta was opened longitudinally, fixed with 4% paraformaldehyde, and photographed with a digital camera (Nikon Coolpix 990). Atherosclerotic lesions were identified as discrete raised white area stained with oil red O. Lesion areas were quantified with IMAGEPRO PLUS. Data are expressed as the percentage of atherosclerotic area relative to the whole aortic area.

### Statistical Analysis

All data are presented as the means ± SD. Results were analyzed by one-way analysis of variance and Student’s t test, using SPSS 13.0 software. Statistical significance was obtained when ρ values were less than 0.05.

## Results

### Betulinic Acid Promoted ABCA1 Expression and Cholesterol Efflux in LPS-treated Macrophages

We first analyzed whether betulinic acid influenced the expression of ABCA1 and cholesterol efflux in THP-1 macrophage-derived foam cells. Our data showed that incubation of cells with betulinic acid in the absence of LPS did not alter ABCA1 expression and apoA-I mediated cholesterol efflux (Fig. S1).

**Figure 1:**
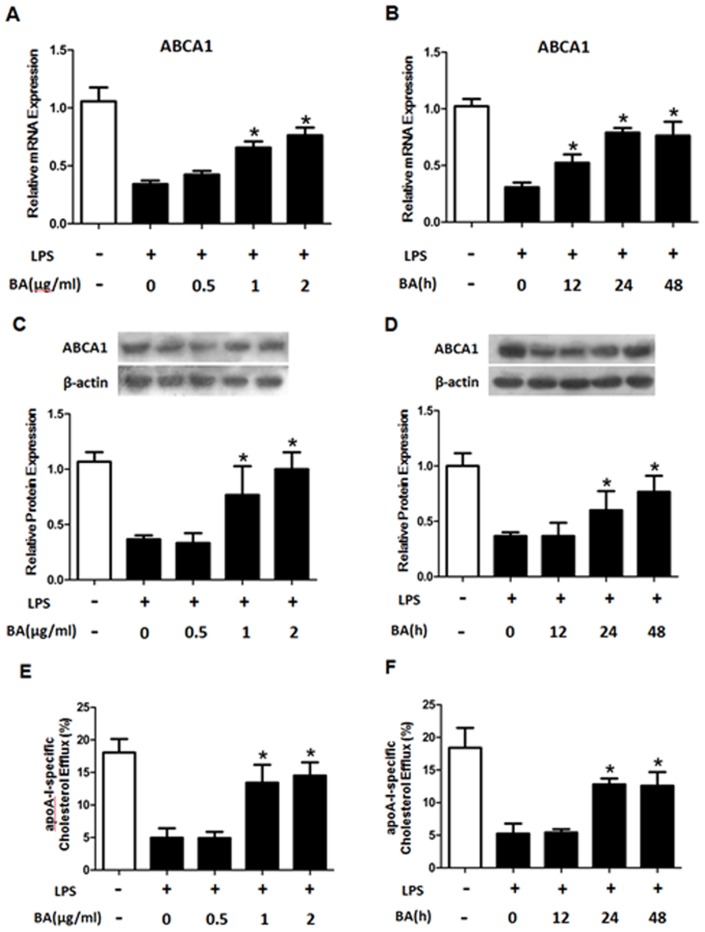
The effect of betulinic acid on the expression of ABCA1 and cholesterol efflux in LPS-treated macrophages. THP-1 macrophage-derived foam cells were cultured in medium containing with or without LPS (10 ng/ml) for 24 h. LPS groups were the cells pre-treated with BA (0, 0.5, 1, 2 µg/ml) for 24 h or with BA (1 µg/ml) for 0, 12, 24 and 48 h, and then exposed to LPS. (A, B, C and D) The levels of ABCA1 mRNA and protein were measured by real-time PCR and Western blotting assays, respectively. (E and F) Cellular cholesterol efflux was analyzed by liquid scintillation counting assays as described. All the results are expressed as mean ± SD. from three independent experiments, each performed in triplicate. *, P<0.05 vs LPS group.

LPS was recently shown to involve in the down-regulation of ABCA1 and the formation of foam cell [19]. Given that BA inhibits LPS-induced inflammation, we tested if BA affected the inhibitory effect of BA on ABCA1 expression. As shown in Fig. 1A–1D, BA decreased the levels of ABCA1 mRNA and protein expression in a concentration- and time-dependent manner. Furthermore, BA treatment decreased cellular cholesterol content in THP-1 macrophage-derived foam cells (Table 1 and Table 2), but increased cellular cholesterol efflux (Fig. 1E–1F).

**Table 1:** Effect of different concentrations of BA on cholesterol content in THP-1 macrophage-derived foam cells.

LPS (10 ng/ml)	−	+		+		+		+		
BA (µg/ml)	−	0		0.5		1		2		
**TC (mg/g)**	480±21		695±31*		623±25		557±19#		531±32#	
**FC (mg/g)**	195±15		269±9*		238±12		231±11		214±21#	
**CE (mg/g)**	285±17		426±23*		385±19		326±14#		317±17#	
**CE/TC(%)**	59.4		61.3		61.8		58.5		59.7	

THP-1 macrophage-derived foam cells were divided into five groups and cultured in medium at 37°C containing with or without LPS (10 ng/ml) for 24 h. LPS groups were the cells pre-treated with BA (0, 0.5, 1, 2 µg/ml) for 24 h, and then exposed to LPS. Cellular cholesterol and cholesterol ester were extracted as described. HPLC was performed to determine cellular levels of total cholesterol (TC), free cholesterol (FC) and cholesterol ester (CE). The results are expressed as the mean ± S.D. of three independent experiments, each performed in triplicate.

* ,p<0.05, vs. control group.

# ,P<0.05 vs. (0 µg/ml) BA group.

**Table 2:** Effect of BA on cholesterol content in THP-1 macrophage-derived foam cells at different time points.

LPS (10 ng/ml)	−		+		+		+		+	
BA (1 µg/ml)	−		0 h		12 h		24 h		48 h	
**TC (mg/g)**	525±27	711±38*		663±29		594±15#		581±32#		
**FC (mg/g)**	328±23	298±19		277±16		235±18#		234±18#		
**CE (mg/g)**	307±15	413±26*		386±21		359±24#		347±11#		
**CE/TC(%)**	58.5	58.1		58.2		60.4		59.7		

THP-1 macrophage-derived foam cells were divided into five groups and cultured in medium at 37°C containing with or without LPS (10 ng/ml) for 24 h. LPS groups were the cells pre-treated with BA (1 µg/ml) for 0, 12, 24 and 48 h, respectively, and then exposed to LPS. Cellular cholesterol and cholesterol ester were extracted as described above. HPLC was performed to determine cellular total cholesterol (TC), free cholesterol (FC) and cholesterol ester (CE). The results are expressed as the mean ± S.D. of three independent experiments, each performed in triplicate.

* ,p<0.05, vs. control group.

# ,P<0.05 vs. (0 µg/ml) BA group.

### Betulinic Acid Inhibited miR-33s Expression

**Figure 2:**
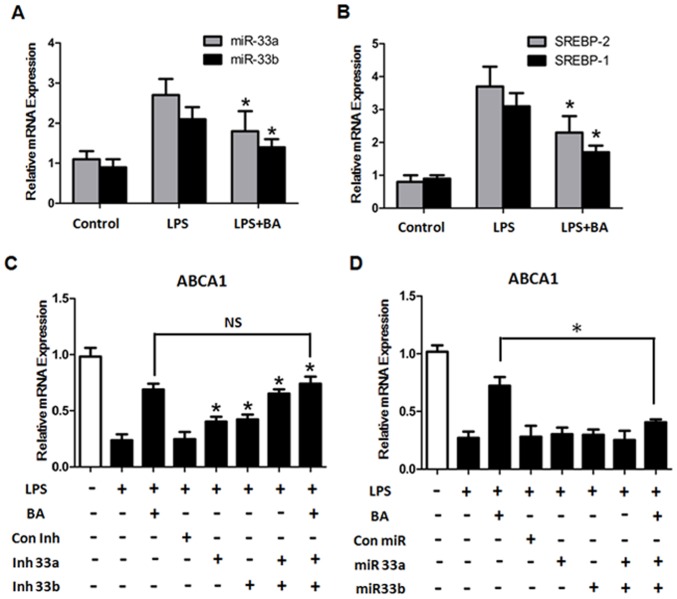
The involvement of miR-33s in the betulinic acid-mediated increase of ABCA1 expression in LPS-treated macrophages. (A and B) THP-1 macrophage-derived foam cells were divided into 3 groups and treated with LPS (10 ng/ml) and/or BA (1 µg/ml) for 24 h, respectively. The expression of miR-33s and SREBPs mRNA was measured by RT-PCR. (C and D) RT-PCR analysis of ABCA1 in THP-1 macrophages transfected with miR-33a/b mimic or anti-miR-33a/b. All the results are expressed as mean ± SD. from three independent experiments, each performed in triplicate. *, P<0.05 vs LPS group.

Recent studies have shown that miR-33 produced from an intron of SREBP inhibits cholesterol efflux by down-regulating ABCA1 expression [20]. To investigate the mechanisms by which BA regulates ABCA1 levels, we examined the mRNA levels of miR-33a/b and their host genes SREBP-2/SREBP-1. As shown in Fig. 2A–2B, LPS significantly increased the levels of miR-33s and SREBPs expression in THP-1 macrophage-derived foam cells, whereas treatment with BA downregulated the expression of miR-33s and SREBPs when compared with LPS only treatment. We also analyzed other miR-33 target genes and found that LPS treatment resulted in a significant decrease in the levels of several of miR-33 target genes involved in cellular cholesterol efflux (NPC1), fatty acid oxidation (CROT and CPT1A) and glucose metabolism (IRS2) (Fig. S2).

To further confirm the functional role of miR-33s in the effect of BA on ABCA1 expression, we assessed the effects of gain and loss of functions of miR-33s on ABCA1 expression in THP-1 cells. The effects of LPS on ABCA1 were blocked by miR-33a/b inhibitor (anti-miR-33a/b) (Fig. 2C). However, ABCA1 levels were not changed significantly (p>0.05) in THP-1 cells transfected with excess wild-type human miR-33a/b mimic oligonucleotides when compared to the LPS group (Fig. 2D), indicating that the endogenous miR-33s levels in LPS-treated cells were sufficient enough to suppress ABCA1 expression. In addition, the effect of BA on ABCA1 expression was inhibited by miR-33a/b mimic (Fig. 2D). Taken together, these findings suggest that BA regulates ABCA1 expression via inhibiting miR-33s levels.

### Betulinic Acid Inhibited Activation of NF-kappaB

**Figure 3:**
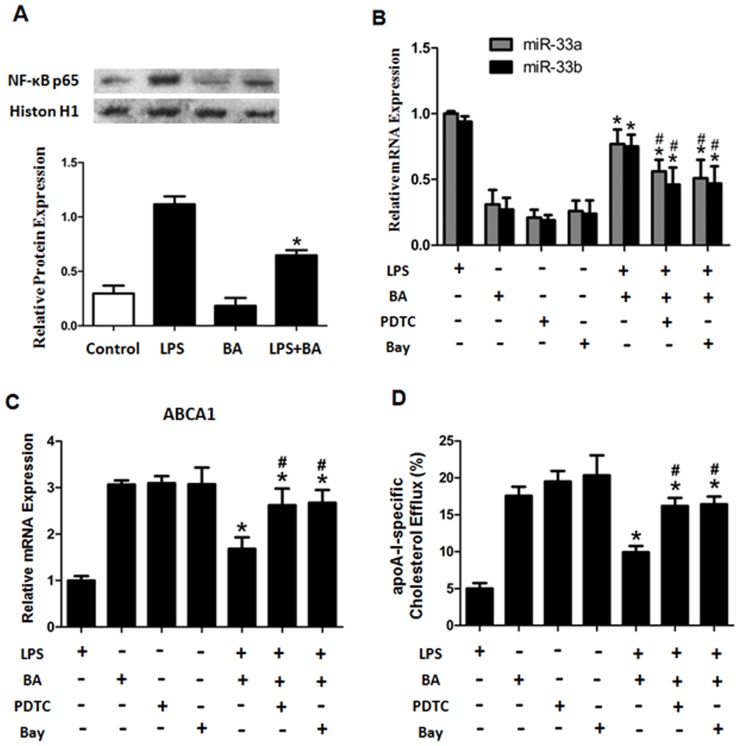
The effect of betulinic acid on NF-κB nuclear protein translocation. (A) THP-1 macrophage-derived foam cells were divided into 3 groups and treated with LPS (10 ng/ml) and/or BA (1 µg/ml) for 24 h, respectively. Protein levels of NF-κB p65 in nuclear extracts were measured and analyzed. (B and C) THP-1 macrophage-derived foam cells were pretreated with PDTC (50 µM) or Bay 11-7082 (5 µM) for 24 h, and the cells were then incubated with LPS for another 24 h with or without pretreatment of BA. The mRNA levels of miR-33s (B) and ABCA1 (C) were confirmed by RT-PCR. (D) Cellular cholesterol efflux was analyzed by liquid scintillation counting assays as shown above. All the results are expressed as mean ± SD. from three independent experiments, each performed in triplicate. *, P<0.05 vs LPS group. ^#^, P<0.05 vs. LPS+BA group.

Activation of the nuclear transcription factor NF-κB plays a key role in pro-inflammatory signaling induced by LPS; therefore, we investigated the effect of BA on NF-κB activation. BA treatment effectively abrogated the promoting effects of LPS on nuclear NF-κB p65 protein levels (Fig. 3A). We next examined the nuclear expression of NF-κB p65 to define the potential effect of NF-κB activation on miR-33s expression. When compared with LPS-stimulated macrophages, BA inhibited miR-33s expression; addition of NF-κB specific inhibitor (PDTC or Bay 11-7082) to BA treatment significantly reduced miR-33s expression in the cells (Fig. 3B), and remarkably promoted ABCA1 expression and cellular cholesterol efflux (Fig. 3C–3D). Together, these results suggest that the decreased NF-kB activity appears to contribute to the inhibitory effect of BA on LPS-induced macrophage miR-33s expression.

### Betulinic Acid Inhibited NF-κB Signaling Pathway in LPS-treated Macrophages

**Figure 4:**
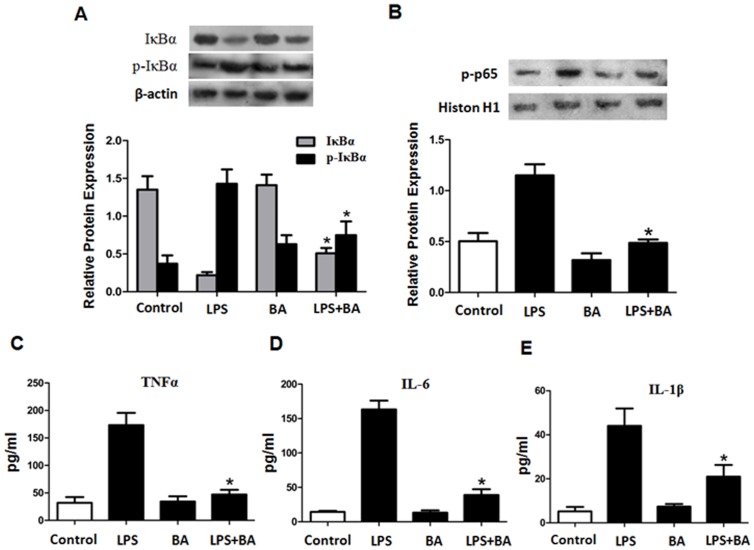
The effect of betulinic acid on NF-κB signaling pathway in LPS-treated macrophages. (A) THP-1 macrophage-derived foam cells were divided into 3 groups and treated with LPS (10 ng/ml) and/or BA (1 µg/ml) for 24 h, respectively. The protein levels of IκBα and p-IκBα in the cytosol were measured and analyzed. (B) Nuclear extracts were prepared and analyzed by Western blot analysis using a specific anti-phospho-p65 Ab. (C, D and E) TNF-α, IL-6 and IL-1β released into the medium were measured by ELISA. All the results are expressed as mean ± SD. from three independent experiments, each performed in triplicate. *, P<0.05 vs LPS group.

The translocation of NF-κB to the nucleus is preceded by phosphorylation, ubiquitination, and proteolytic degradation of IκBα. To determine whether inhibition of NF-κB activation by BA was mediated by IκBα, we examined IκBα status in the cytoplasm by Western blot analysis. As shown in Fig. 4A, BA treated cells showed a significant reduction in the levels of phosphorylated IκBα (p-IκBα), which was correlated with exclusively decreased nucleus accumulation of NF-κB p65 protein (Fig. 3A). In addition to stimulus-induced nuclear translocation of NF-κB, several lines of evidence suggest that stimulus-induced phosphorylation of the p65 subunit plays a key role in the transcriptional activation of NF-κB after the nuclear translocation. Thus, we characterized the phosphorylation of p65 using a specific anti-phospho-p65 antibody. As shown in Fig. 4B, the phosphorylation of p65 induced by LPS was suppressed by addition of BA. We then examined the effect of BA on LPS-stimulated production of inflammatory cytokines in macrophages. As shown in Fig. 4C–4E, BA significantly inhibited LPS-stimulated secretion of TNFα, IL-6 and IL-1β. Taken together, our findings showthat BA inhibits NF-κB signaling pathway in LPS-treated macrophages most likely via inhibiting IκBα and NF-κB phosphorylation.

### Betulinic Acid Reduced Atherosclerotic Lesions in apoE^−/−^ Mice

**Figure 5:**
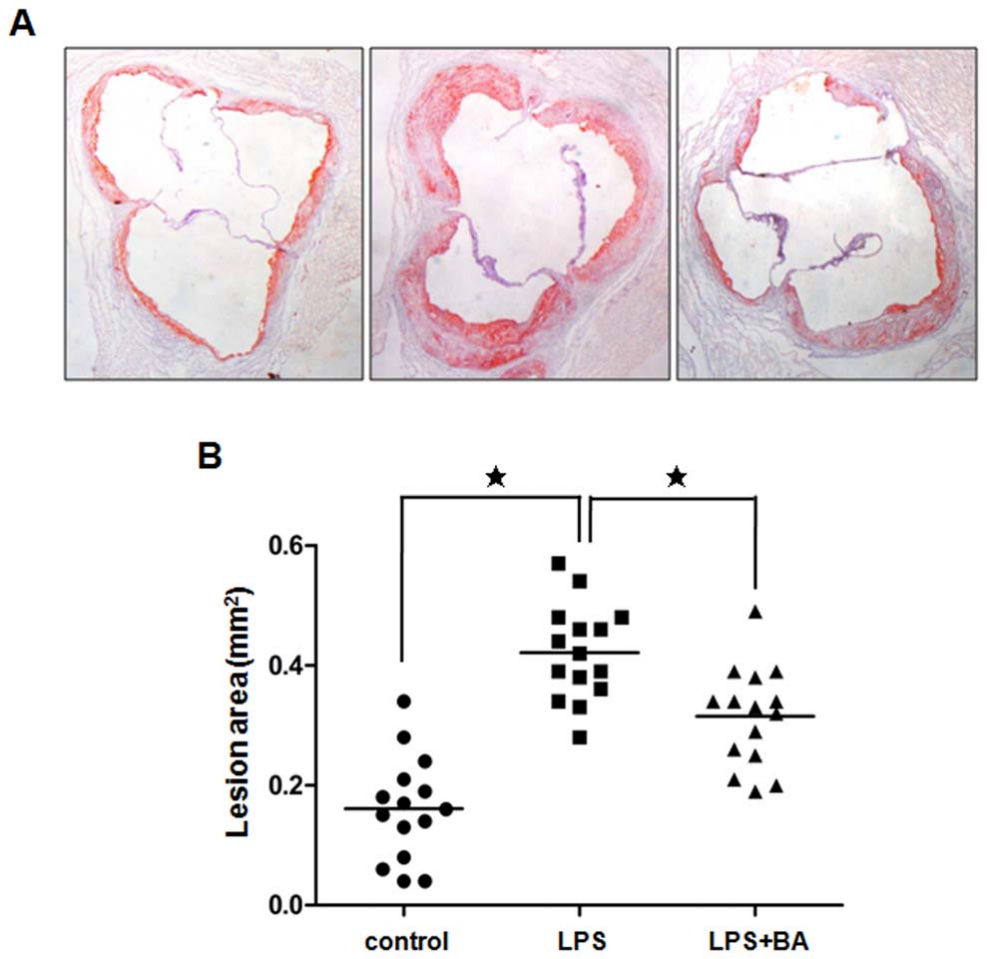
The effect of betulinic acid on atherosclerotic lesions in apoE^−/−^ mice. 8-week-old male apoE^−/−^ mice were intraperitoneally with PBS, LPS (2.5 mg/kg body wt) or LPS (2.5 mg/kg body wt) plus administrated with BA (50 mg/kg body wt) once a week for 8 weeks. (A) Representative Oil-red-O staining of aortic sinus lesion. Original magnification: ×40. (B) Quantification of the lesion area of mice (n = 15/group). Each symbol indicates data obtained from a single mouse. Data are the mean ± SEM. * P<0.05.

BA has been used successfully to prevent abdominal fat accumulation in mice with no apparent toxicity [10]. As increased ABCA1 expression and decreased NF-kB activity can reduce atherosclerotic lesion sizes, we measured the effects of BA on the development of atherosclerosis in apoE^−/−^ mice. Consistent with previous findings (reference), LPS-challenged apoE^−/−^ mice showed significantly increased lesion sizes compared to PBS-treated animals. BA treatment led to a significant reduction in lesion sizes in LPS-injected apoE^−/−^ mice (Fig. 5A–5B, and fig. S3). It has been reported that infection and inflammation are often accompanied by an increase in serum triglyceride (TG) levels in all species including mice and in serum total cholesterol (TC) levels in rodents [21]. Thus, we evaluated these parameters in apoE^−/−^ mice (Table 3). The results showed that both TG and TC levels in animals treated with BA were decreased. Further detailed analysis of the plasma lipoproteins revealed an increase in HDL cholesterol and a decrease in LDL cholesterol levels in the BA-treated group, suggesting that BA-induced atherosclerosis regression is at least partially through regulating the levels of plasma lipoprotein cholesterol. The results are in line with our *in vitro* results that BA impairs LPS-induced reduction in ABCA1 expression.

**Table 3:** Body weight and plasma lipid profile in apoE^−/−^ mice.

	Control (n = 15)	LPS (n = 15)	LPS+BA (n = 15)
Body weight (g)	29.57±3.14	32.16±2.89	31.41±3.58
TG (mmol/L)	0.48±0.11	1.25±0.26*	0.95±0.23#
TC (mmol/L)	9.51±1.34	13.95±2.65*	9.15±2.16#
HDL-C (mmol/L)	1.99±0.53	1.05±0.17*	1.74±0.19#
LDL-C (mmol/L)	7.51±1.15	12.89±2.63*	7.36±0.84#

Plasma from different experimental groups was measured enzymatically. The data were the means ± SEM from the indicated numbers of male apoE^−/−^ mice in each group.

* ,P<0.05 vs control.

# ,P<0.05 vs. LPS group.

### Betulinic Acid Regulated miR-33s/ABCA1 Expression and NF-κB Signaling Pathway *in vivo*

**Figure 6:**
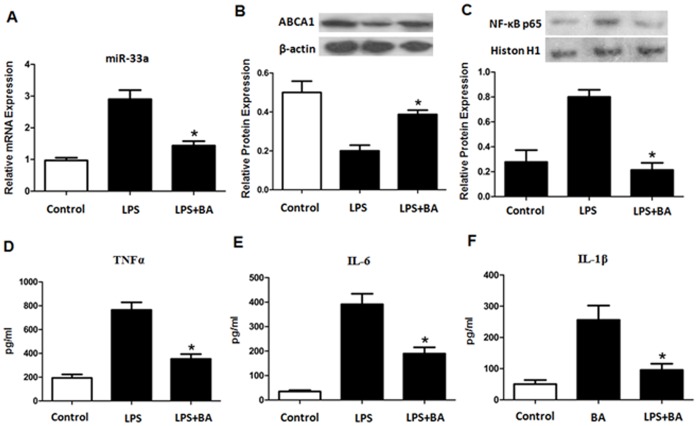
The effect of betulinic acid on miR-33a/ABCA1 expression and NF-κB activation in apoE^−/−^ mice. 8-week-old male apoE^−/−^ mice were randomly divided into three groups as described in materials and methods. (A) Expression of miR-33a mRNA in the aorta was confirmed by RT-PCR. (B and C) Western blot of aorta ABCA1 protein (B) and aorta NF-κB p65 protein (C). (D, E and F) Plasma levels of TNF-α, IL-6 and IL-1β. All the results are expressed as mean ± SD. *, P<0.05 vs LPS group.

Based on our *in vitro* and *in vivo* observations, we examined whether BA could regulate miR-33s/ABCA1 expression *in vivo*. As shown in Fig. 6A–6B, BA significantly reduced the expression of miR-33a and promoted the expression of ABCA1 as compared with LPS alone treatment, suggesting that the elevation in plasma HDL-C and regression of atherosclerotic lesions induced by BA may be contributed by the increased levels of ABCA1. In addition, we observed that BA suppressed the activation of NF-κB and decreased the plasma pro-inflammatory cytokines levels (Fig. 6C–6F). The results are in line with our *in vitro* observations on the protective effects of BA and strengthen our hypothesis that BA can block NF-κB-miR-33s-ABCA1 cascades triggered by LPS *in vivo*.

## Discussion

**Figure 7:**
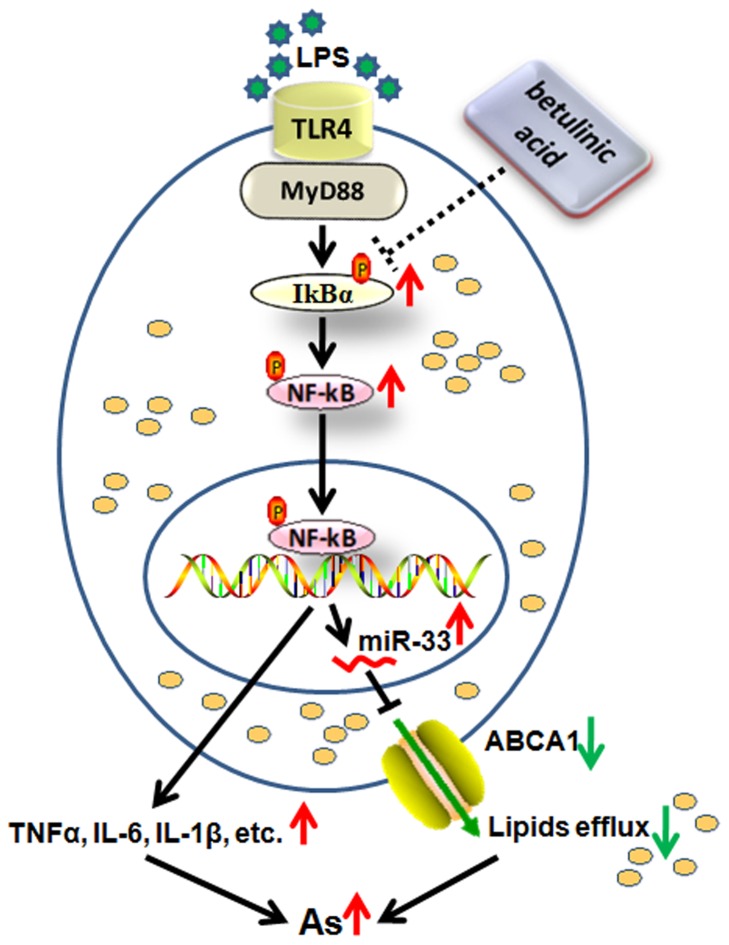
Schematic representation of the effect of betulinic acid on NF-κB pathway and miR-33s/ABCA1-dependent lipid efflux in macrophages. The results of the present study revealed the following possible mechanisms: NF-κB activation stimulated by LPS directly promotes the expression of miR-33s and inflammatory cytokine production. miR-33s repress the expression of ABCA1 and subsequently decrease the efflux of cholesterol and phospholipids. Inhibition of the efflux of macrophage lipids and promotion of inflammatory cytokine release lead to increased atherosclerosis. BA treatment decreases the phospho-protein levels of IκBα and the subsequent NF-κB activation. Inhibition of NF-κB activation suppresses miR-33s expression and promotes ABCA1-dependent lipid efflux in macrophages. In addition, BA possesses an anti-inflammatory activity by reducing the levels of TNF-α, IL-6 and IL-1β. Together, BA plays an important role in the reduction of atherosclerotic lesions. →: Activation; ┤: inhibition.

In this study, we investigated the molecular mechanisms of BA-induced reductions in atherosclerotic lesions. As illustrated in Fig. 7, our results show that BA, a triterpenoid belonging to lupane series, induces cholesterol efflux through promoting ABCA1 expression in macrophages through inhibiting the NF-κB/miR-33s signaling cascade. Moreover, BA effectively attenuates NF-κB-mediated inflammatory cytokine release.

Macrophage-derived cholesteryl ester-rich foam cells develop within the arterial wall as a result of excessive internalization of lipoproteins, which subsequently promotes early atherosclerotic plaque formation [22]. Maintenance of cellular cholesterol homeostasis is important for normal human physiology. ABCA1, a key factor in cholesterol homeostasis, mediates the efflux of cellular free cholesterol and phospholipids to an extracellular acceptor, apoA-I, to form nascent HDL. Macrophage-specific deletion of ABCA1 results in an increase in atherosclerosis, whereas overexpression of ABCA1 in macrophages provides protection from atherosclerosis [23]. In this study, our data provide a new mechanism of the protective effect of BA on the development of cardiovascular diseases. We found that BA upregulated the expression of ABCA1 and promoted cellular cholesterol efflux in THP-1 macrophage-derived foam cells. In addition, cellular cholesterol and cholesterol ester content were decreased when the cells were treated with BA. Our data suggest that the BA-induced increase in free cholesterol efflux from THP-1 macrophage-derived foam cells is at least mainly, if not completely dependent on enhanced ABCA1-mediated cholesterol efflux.

MicroRNAs are short 21–24-nucleotide long, nonprotein-coding RNAs that are important regulators of gene expression. By binding to the 3′ untranslated region of protein-coding mRNA transcripts, they can reduce protein translation from these transcripts and in some cases lead to mRNA degradation [24]. Several diseases have been reported to be associated with dysregulated miRNA expression. miR-146a and miR-155 have been implicated in the development of rheumatoid arthritis, likely via regulating components of the inflammatory response [25], [26]. Jennewein *et al*. also observed that induction of miR-27b is partially NF-κB-dependent [27]. There are two isoforms of human miR-33s, miR-33a and miR-33b present on introns of the SREBP-2 and SREBP-1genes, respectively. Rodents only express one isoform, the homologue of the human miR-33a. Manipulations of miR-33s using silencing and overexpression approaches show that these small miRNAs repress ABCA1 mRNA and protein expression as well as cholesterol export from human and murine cells [14]. However, most studies have been focused on how miR-33s regulate the expression of predicted target genes. Very few reports study the regulation of miR-33s in cells. MiR-33s are co-regulated with SREBPs. Thus, molecules that regulate SREBPs can modulate miR-33 production, such as cellular levels of cholesterol and oxysterols. We showed here for the first time that NF-kB positively regulated the expression of miR-33s. BA reduces NF-κB levels, thereby reducing miR-33s, increasing ABCA1 levels and subsequently enhancing cholesterol efflux from macrophages. Given that over 1000 human miRNAs have been identified, rendering miRNAs one of the most abundant classes of regulatory molecules, deciphering their biological functions in NF-κB dysregulation is essential to appreciate the complexity of immune systems and to develop therapeutics against atherosclerotic cardiovascular disease and other related diseases.

Inflammation is recognized as a major mechanism in the formation and development of atherosclerotic lesion. NF-κB, a major transcription factor in inflammatory responses, is involved in the regulation of genes involved in inflammatory, apoptosis, and cell proliferation [28]. Many NF-κB inducers and regulated genes have been implicated directly or indirectly in atherosclerosis. The discovery of natural or pharmaceutical, selective and specific inhibitors of NF-κB pathway may ultimately prove to be a promising anti-atherosclerotic, anti-inflammatory, antiangiogenic and antiapoptotic therapeutic instrument that could potentially reduce inflammation, attenuating atherogenesis and preventing its complications [29]. Our study in apoE^−/−^ mice showed that BA inhibited NF-κB activation and increased circulating HDL-C, reduced TC and LDL-C, and regressed established atherosclerosis. These findings indicate that BA might be a promising clinical approach for raising HDL and reducing inflammation in the treatment of cardiovascular disease.

NF-κB is maintained in a latent form in the cytoplasm, where it exists in complex with IkBs. Upon the kinase-dependent phosphorylation and subsequent ubiquitination and degradation of IκB, free NF-κB is translocated to the nucleus where it binds to the consensus sequence of pro-inflammatory genes and evokes its expression [30]. BA inhibits the activation of LPS and other inflammatory agents in many cell types. The inhibitory activity correlates with the suppression of LPS-induced IκB phosphorylation and degradation, p65 phosphorylation and nuclear translocation, and the transcription of NF-κB-targeted genes. Various ways may account for the inhibitory effect of BA on NF-κB activation. However, BA most likely suppresses a common step in the pathways of NF-κB activation since it inhibits NF-κB activation induced by highly diverse stimuli, including cigarette smoke, TNFα, H_2_O_2_, LPS, PMA, and IL-1 [11], [31], [32]. In response to these stimuli, NF-κB activation requires sequential phosphorylation at serine residues at positions 32 and 36 of IκB. In this study, we showed that BA blocked LPS-induced phosphorylation of IκB, suggesting that the inhibition effect BA on NF-κB is universal and reliability.

In conclusion, this study provides novel insights into the protective effect of BA on enhancing cholesterol efflux via up-regulating ABCA1 expression, which is mediated by inhibiting NF-κB signaling pathway and miR-33 expression. In addition, BA promotes circulatory HDL-C levels, reduces inflammation levels, and regresses established atherosclerosis in vivo. BA may therefore be a promising therapeutic agent for the prevention of atherosclerotic progression.

## Supporting Information

Figure S1 **Effects of betulinic acid on the expression of ABCA1 and cholesterol efflux in THP-1 macrophage-derived foam cells.** (A and B) ABCA1 mRNA expressions were measured by real-time PCR. (C and D) Cellular cholesterol efflux was analyzed by liquid scintillation counting assays as shown in materials and methods. All the results are expressed as mean ± SD. from three independent experiments, each performed in triplicate.
(TIF)

Figure S2 **Effects of betulinic acid on the expression of NPC1, CROT, CPT1A and IRS2 in LPS-treated macrophages.** The expression of NPC1, CROT, CPT1A and IRS2 mRNA was confirmed by RT-PCR. All the results are expressed as mean ± SD. from three independent experiments, each performed in triplicate. *, P<0.05 vs control group. ^#^, P<0.05 vs. LPS group.
(TIF)

Figure S3 **Betulinic acid treatment decreased atherosclerosis plaque in apoE^−/−^ mice.** 8-week-old male apoE^−/−^ mice were intraperitoneally with PBS, LPS (2.5 mg/kg body wt) or LPS (2.5 mg/kg body wt) plus administrated with BA (50 mg/kg body wt) once a week for 8 weeks. (A) Representative Oil-red-O staining of en face aortas. Original magnification: ×40. (B) Atherosclerotic area is expressed as a percentage relative to the whole aortic area (n = 15/group). Columns represent the mean ± SEM of 6 mice. *, P<0.05 vs control group. ^#^, P<0.05 vs. LPS group.
(TIF)
