# Supplementary figures and images for: Antagonism of Betulinic Acid on LPS-Mediated Inhibition of ABCA1 and Cholesterol Efflux through Inhibiting Nuclear Factor-kappaB Signaling Pathway and miR-33 Expression
Source: PLoS One. 2013 Sep 25;8(9):e74782. doi: 10.1371/journal.pone.0074782 (PMC3783495; doi:10.1371/journal.pone.0074782)

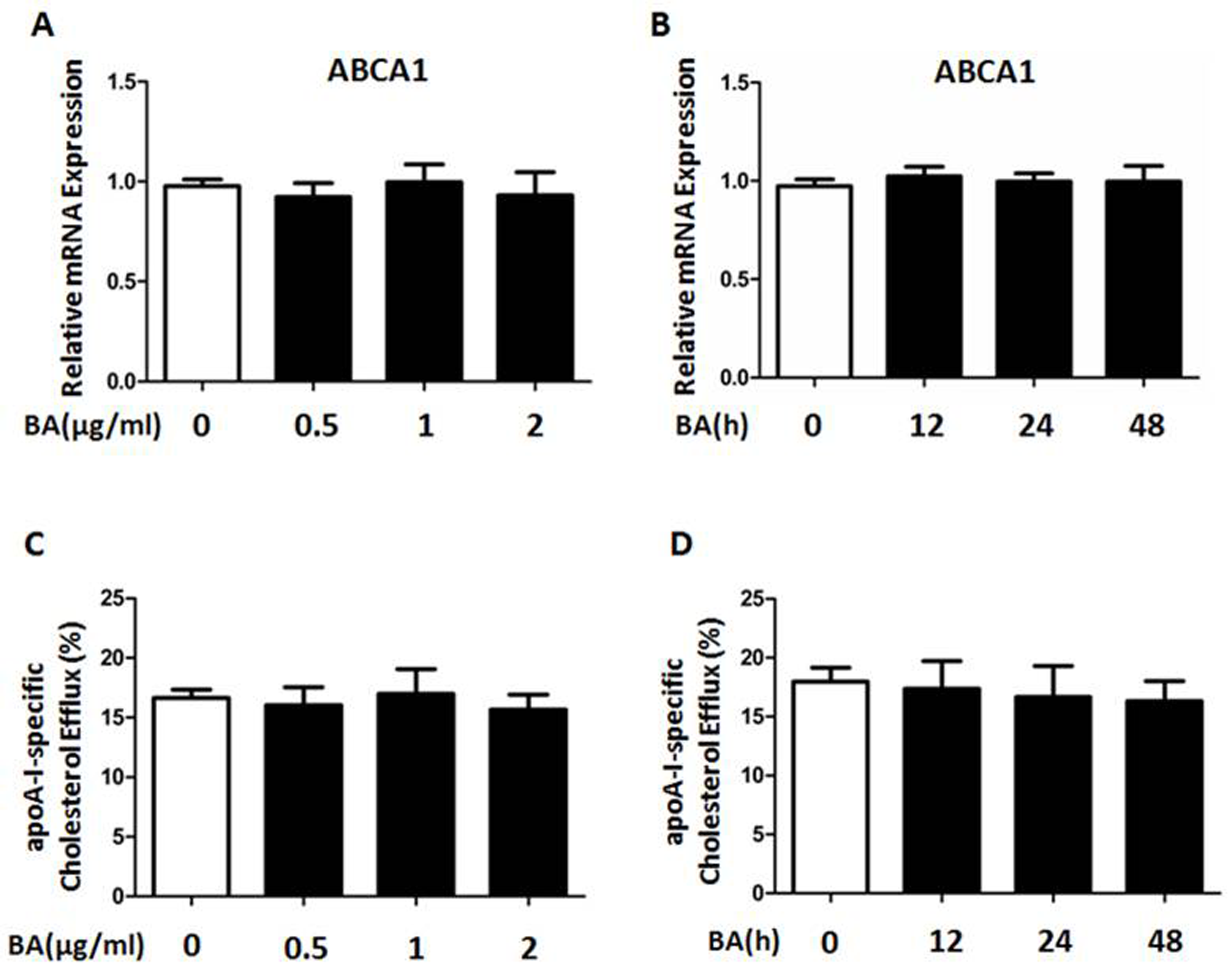

Supplement: Figure S1 — Effects of betulinic acid on the expression of ABCA1 and cholesterol efflux in THP-1 macrophage-derived foam cells. (A and B) ABCA1 mRNA expressions were measured by real-time PCR. (C and D) Cellular cholesterol efflux was analyzed by liquid scintillation counting assays as shown in materials and methods. All the results are expressed as mean ± SD. from three independent experiments, each performed in triplicate. (TIF) [file pone.0074782.s001.tif]

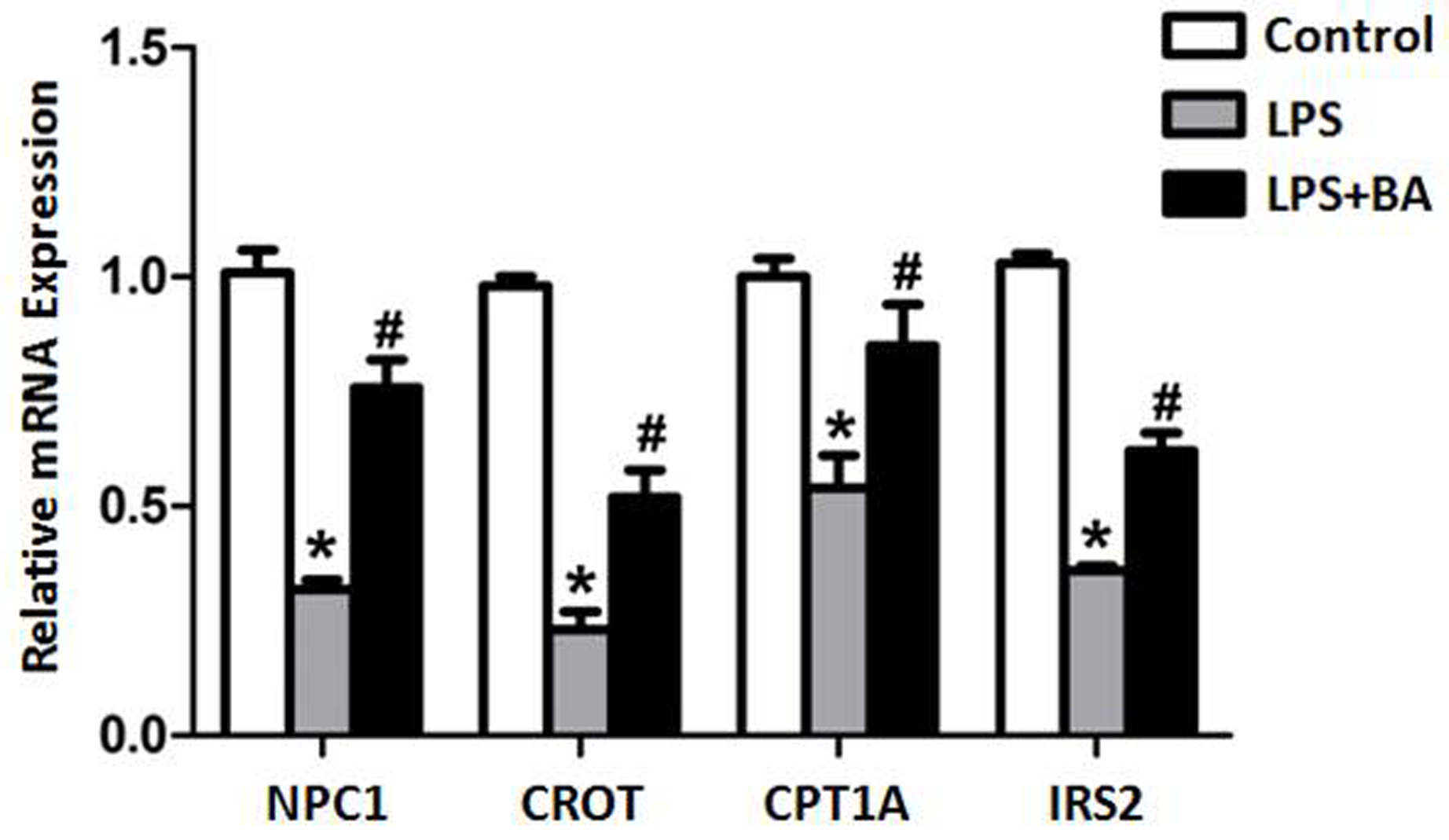

Supplement: Figure S2 — Effects of betulinic acid on the expression of NPC1, CROT, CPT1A and IRS2 in LPS-treated macrophages. The expression of NPC1, CROT, CPT1A and IRS2 mRNA was confirmed by RT-PCR. All the results are expressed as mean ± SD. from three independent experiments, each performed in triplicate. *, P<0.05 vs control group. #, P<0.05 vs. LPS group. (TIF) [file pone.0074782.s002.tif]

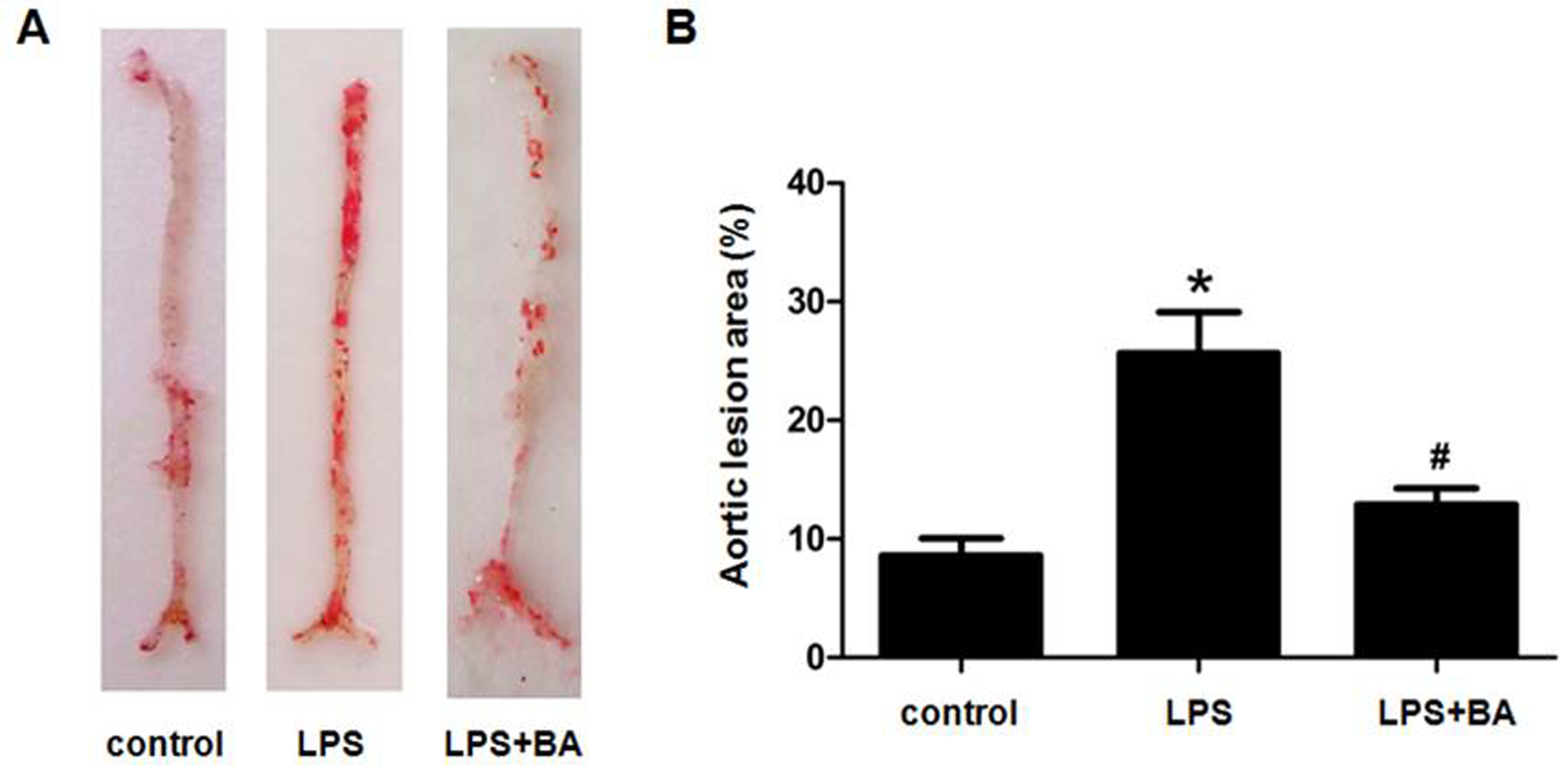

Supplement: Figure S3 — Betulinic acid treatment decreased atherosclerosis plaque in apoE−/− mice. 8-week-old male apoE−/− mice were intraperitoneally with PBS, LPS (2.5 mg/kg body wt) or LPS (2.5 mg/kg body wt) plus administrated with BA (50 mg/kg body wt) once a week for 8 weeks. (A) Representative Oil-red-O staining of en face aortas. Original magnification: ×40. (B) Atherosclerotic area is expressed as a percentage relative to the whole aortic area (n = 15/group). Columns represent the mean ± SEM of 6 mice. *, P<0.05 vs control group. #, P<0.05 vs. LPS group. (TIF) [file pone.0074782.s003.tif]
